# A dataset for automatic violence detection in videos

**DOI:** 10.1016/j.dib.2020.106587

**Published:** 2020-11-26

**Authors:** Miriana Bianculli, Nicola Falcionelli, Paolo Sernani, Selene Tomassini, Paolo Contardo, Mara Lombardi, Aldo Franco Dragoni

**Affiliations:** a Università degli Studi di Roma La Sapienza, Piazzale Aldo Moro 5, Roma 00185, Italy; b Dipartimento di Ingegneria dell'Informazione, Università Politecnica delle Marche, Via Brecce Bianche 12, Ancona 60131, Italy; c Gabinetto Interregionale di Polizia Scientifica per le Marche e l'Abruzzo, Via Gervasoni 19, Ancona 60129, Italy

**Keywords:** Violence detection, Crime detection, Computer vision, Deep learning

## Abstract

The automatic detection of violence and crimes in videos is gaining attention, specifically as a tool to unburden security officers and authorities from the need to watch hours of footages to identify event lasting few seconds. So far, most of the available datasets was composed of few clips, in low resolution, often built on too specific cases (e.g. hockey fight). While high resolution datasets are emerging, there is still the need of datasets to test the robustness of violence detection techniques to false positives, due to behaviours which might resemble violent actions. To this end, we propose a dataset composed of 350 clips (MP4 video files, 1920 × 1080 pixels, 30 fps), labelled as non-violent (120 clips) when representing non-violent behaviours, and violent (230 clips) when representing violent behaviours. In particular, the non-violent clips include behaviours (hugs, claps, exulting, etc.) that can cause false positives in the violence detection task, due to fast movements and the similarity with violent behaviours. The clips were performed by non-professional actors, varying from 2 to 4 per clip.

## Specifications Table

**Table:** 

Subject	Computer Vision and Pattern Recognition
Specific subject area	Violence Detection in Videos
Type of data	Video (mp4), Text files (csv)
How data were acquired	The clips were recorded with two cameras placed in two different spots, building a dataset with videos from two different points of view. The cameras are: • The front camera of the Asus Zenfone Selfie ZD551KL (13 MP, Auto Focus, f/2.2). • The TOPOP Action Cam OD009B (12 MP, fisheye lens 170°).
Data format	Raw
Parameters for data collection	The clips in the dataset are in MP4 format, H.264 codec, with a resolution of 1920 × 1080 pixels, and 30 fps. The average length of the clips is 5.63 s (minimum 2 s, maximum 14 s). 230 clips out of the 350 included in the dataset are labelled as “violent”, whilst the remaining 120 clips are labelled as “non-violent”.
Description of data collection	The dataset includes 350 clips, split into two main directories, “violent” and “non-violent”. Such directories are split into subdirectories, “cam1” and “cam2”: • “violent/cam1” includes 115 clips representing violent behaviours; • “violent/cam2” includes 115 clips with the same violent behaviours in “violent/cam1”, but recorded with a different camera and from a different point of view; • “non-violent/cam1” includes 60 clips representing non-violent behaviours; • “non-violent/cam2” includes 60 clips with the same non-violent behaviours in “non-violent/cam1” but recorded with a different camera and from a different point of view. The clips were performed by a group of non-professional actors (varying from 2 to 4 per clip) simulating violent actions and non-violent actions.
Data source location	Dipartimento di Ingegneria dell'Informazione, Università Politecnica delle Marche, Ancona, Italy.
Data accessibility	Public repository: GitHub (https://github.com) Repository name: A Dataset for Automatic Violence Detection in Videos Direct URL to data: https://github.com/airtlab/A-Dataset-for-Automatic-Violence-Detection-in-Videos

## Value of the Data

• As the interest towards automatic detection of violence and crimes in video is increasing, the clips in the presented dataset are intended to train and benchmark techniques for automatic violence detection in videos.
• In the short and mid-term, researchers can use the Full HD clips as an additional open dataset to train and test their algorithms. In the long-term, law enforcement authorities and the entire community might benefit from fine-tuned algorithms, capable of reducing the decision time in violence and crime detection.
• A specific goal of the dataset is to verify the robustness to false positives of the violence detection techniques. Thus, experiments involving the assessment of the classification accuracy of algorithms can consider this specific feature in the evaluation phase.

## 1 Data Description

The pervasiveness of video surveillance cameras and the need of watching footages and making decisions in a very short time [1] boosted the interest of researchers towards techniques for the automatic detection of violence and crimes in videos. In facts, both techniques based on handcrafted features [2,3] and deep learning [4,5] demonstrated their accuracy for automatic violence detection on open datasets such as the Hockey Fight Dataset [6], the Movie Fight Dataset [6], and the Crowd Violence Dataset [7]. However, such datasets include few low-res videos, sometimes in too specific environments (e.g. hockey arenas). These issues have been faced by the RWF-2000 [8], a dataset including 2000 clips from real video surveillance cameras. Nevertheless, in terms of accuracy, especially for the prevention of false positives, there is still the need to understand the effectiveness of the violence detection techniques in clips showing rapid moves (hugs, claps, high-fives, etc.) which are not violent. To this end, we present a dataset for violence detection specifically designed to include, as non-violent clips, scenes which can cause false positives.

**Fig. 1:**
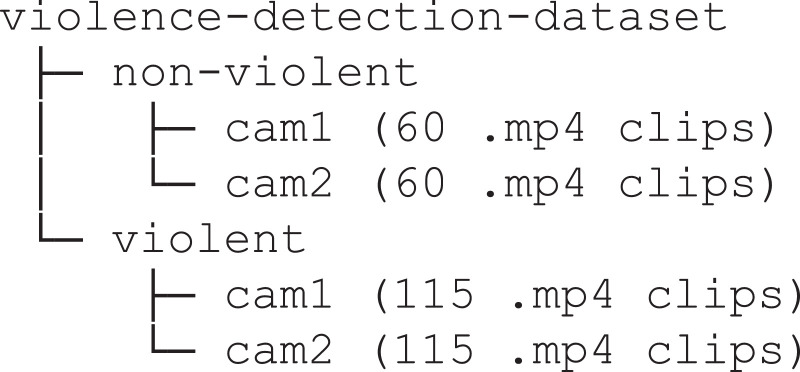
The structure of the data repository with the 350 clips of the dataset, split in non-violent (120 clips) and violent (230 clips).

The dataset is composed of 350 clips which are MP4 video files (H.264 codec) of an average length of 5.63 s, with the shortest video lasting 2 s and the longest 14 s. For all the clips, the resolution is 1920 × 1080 pixels and the frame rate 30 fps. The dataset is organized into directories as shown in Fig. 1.

The dataset is split into two main directories, “non-violent” and “violent”, labelling the included clips as showing non-violent behaviours and violent behaviours respectively. The directories are split into two subdirectories, “cam1” and “cam2”:

• “non-violent/cam1” includes 60 clips representing non-violent behaviours;
• “non-violent/cam2” includes 60 clips with the same non-violent behaviours in “non-violent/cam1” but recorded with a different camera and from a different point of view;
• “violent/cam1” includes 115 clips representing violent behaviours;
• “violent/cam2” includes 115 clips with the same violent behaviours in “violent/cam1” but recorded with a different camera and from a different point of view.

**Fig. 2:**
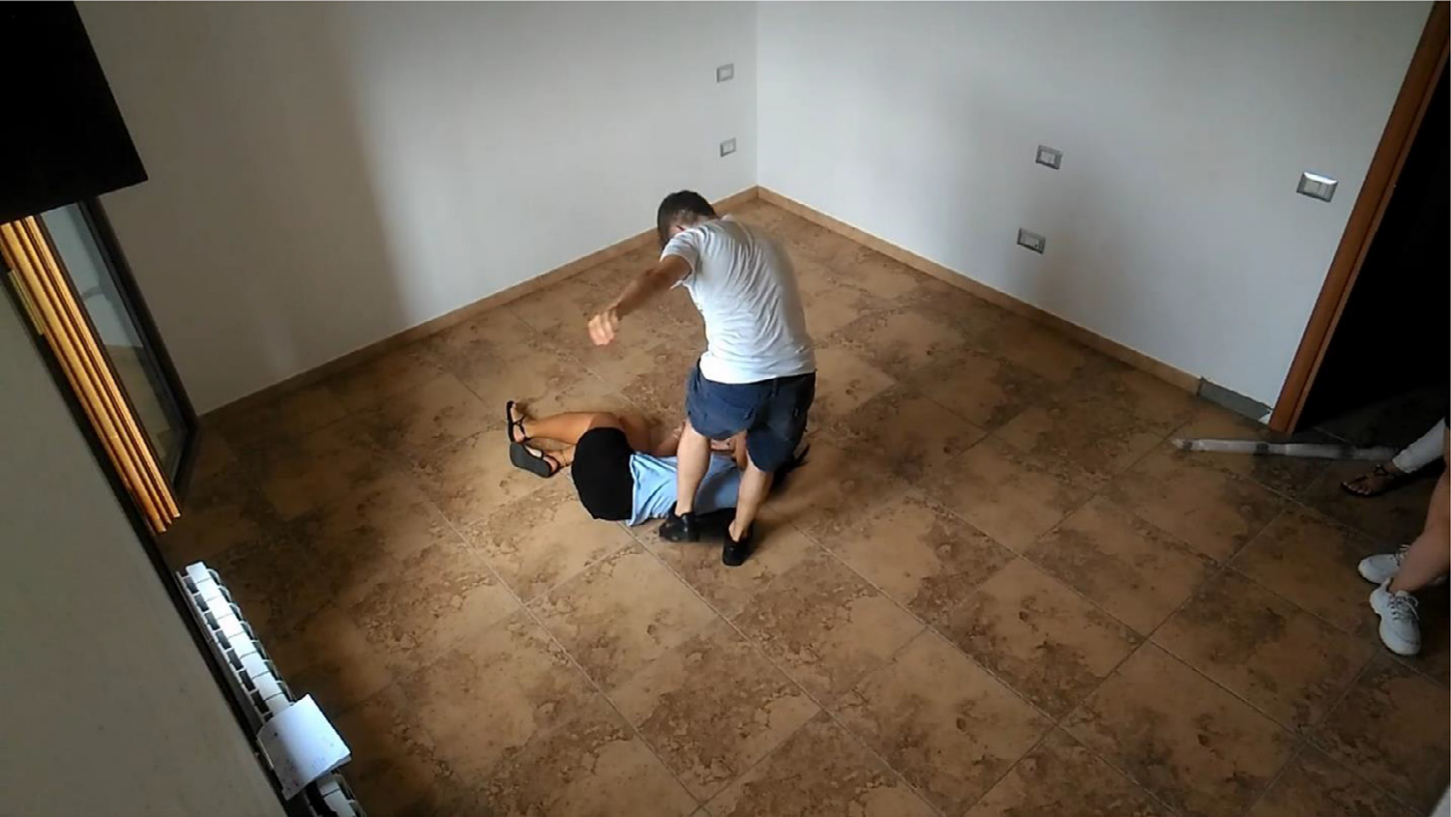
Example of a frame from a violent clip (camera 1).

**Fig. 3:**
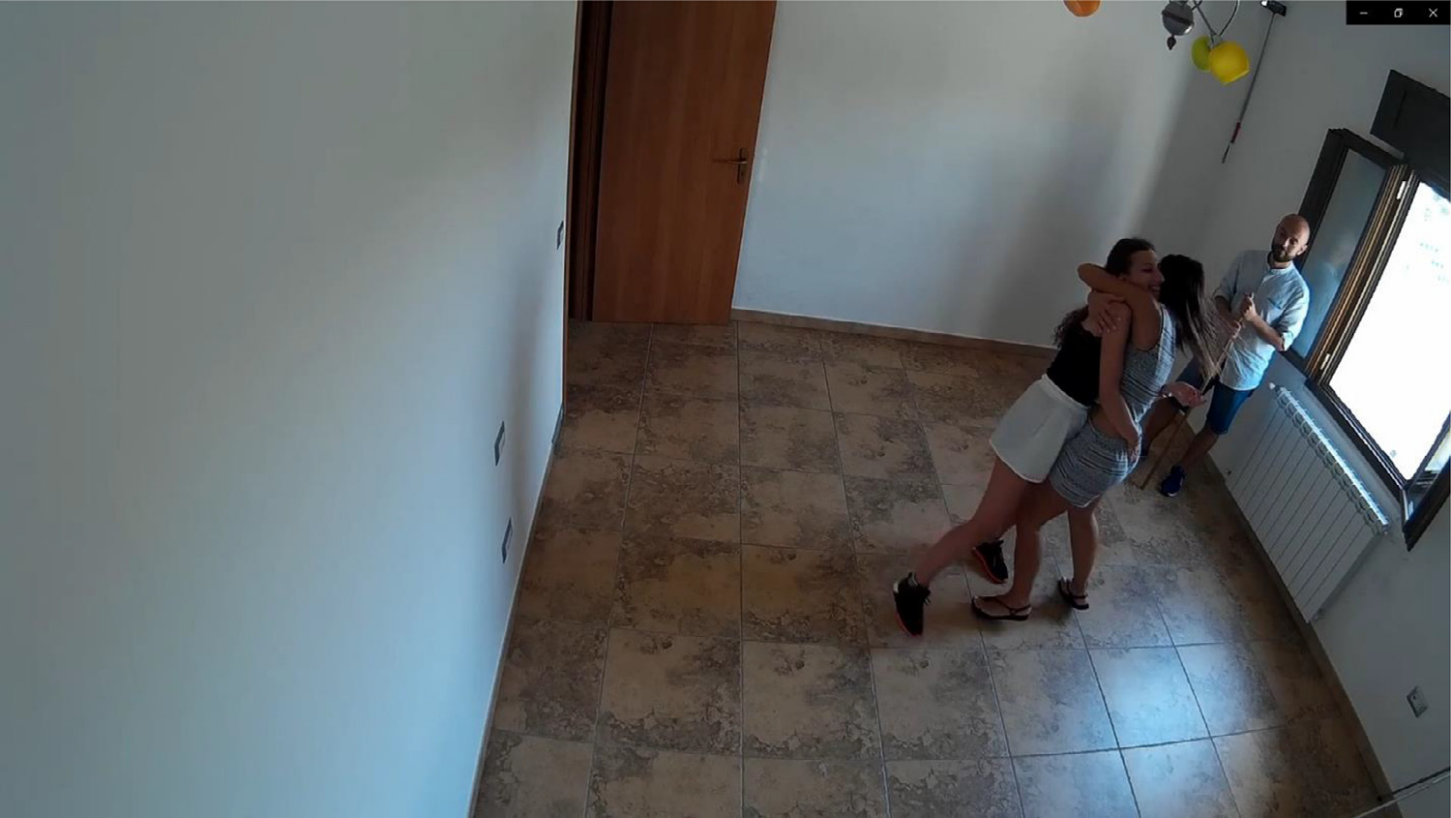
Example of a frame from a non-violent clip (camera 2).

The clips were performed by a group of non-professional actors, varying from 2 to 4 per clip. For the violent clips (Fig. 2), the actors were asked to simulate actions frequent in brawls, such as kicks, punches, slapping, clubbing (beating with a cane), stabbing, and gun shots. For the non-violent clips (Fig. 3), the actors were asked to simulate actions which can result in false positives by violence detection techniques due to the speed of movements or the similarity with violent actions. Specifically, the non-violent clips include actions such as hugging, giving high fives and clapping, exulting, and gesticulating.

An additional labelling is provided in three csv files available in the main data repository directory:

- “action-class-occurrences.csv” lists all the actions recorded in the clips, with the number of times each action occurs in the dataset and a label to explain if the action is violent (y) or not (n). All the actions recorded in the clips are listed in Table 1;

**Table 1:** The list of the recorded actions, with the number of occurrences in the dataset.

Violent actions		Non-violent actions	
Action	# of occurrences	Action	# of occurrences
fight	46	greet	33
club	36	hug	16
punch	23	handgesture	15
push	22	jump	10
kick	21	highfive	6
slap	18	handshake	3
stab	15	walk	1
gunshot	14		
choke	13
- “non-violent-action-class.csv” lists the actions included in each non-violent clip;
- “violent-action-class.csv” lists the actions included in each violent clip.

## 2 Experimental Design, Materials and Methods

As highlighted in a previous study [5], violence detection techniques can fail due to actions and behaviours which are wrongly interpreted as violent, due to fast movements and similarity with violent behaviours. To this end, the non-violent clips were recorded to specifically challenge techniques and prevent false positives, even with datasets unbalanced towards the violent clips, as the one proposed in this paper. For the clips representing violent behaviours, in addition to kicks, punches and slapping, a plastic toy gun, a plastic toy knife, and a wood cane rolled into bubble wrap sheets were used to simulate actions involving weapons such as gun shots, stubbing, and beating.

The clips were recorded with two cameras placed in two different spots, building a dataset with videos from two different points of view. The cameras are:

• The front camera of the Asus Zenfone Selfie ZD551KL (13 MP, Auto Focus, f/2.2).
• The TOPOP Action Cam OD009B (12 MP, fisheye lens 170°).

All the clips were recorded in the same room, with natural lighting conditions. The Asus Zenfone was placed in the top left corner in front of the door, while the Action Cam was placed in the top right corner on the door side. All the performed actions and behaviours were recorded with both cameras. Therefore, all the clips with the same label and name, but in different final directories (for example “non-violent/cam1/1.mp4” and “non-violent/cam2/1.mp4”) represent the same action, recorded from two different perspectives (the “cam1” directory identifies the Asus Zenfone, while the “cam2” directory identifies the Action Cam).

In addition to the main classification of the clips into violent and non-violent, we manually annotated the actions performed in each clip. This annotation can be used for further classification experiments with violence detection techniques, to train and test algorithms capable of performing action recognition.

## Ethics Statement

All the actors involved in the clip recording read and signed an informed consent, conserved at the Artificial Intelligence and Real-Time System Laboratory at the “Dipartimento di Ingegneria dell'Informazione” of “Università Politecnica delle Marche”.

## CRediT authorship contribution statement

**Miriana Bianculli:** Conceptualization, Methodology, Investigation. **Nicola Falcionelli:** Conceptualization, Methodology, Data curation. **Paolo Sernani:** Writing - original draft, Writing - review & editing. **Selene Tomassini:** Writing - review & editing. **Paolo Contardo:** Writing - review & editing. **Mara Lombardi:** Supervision. **Aldo Franco Dragoni:** Supervision.

## Declaration of Competing Interest

The authors declare that they have no known competing financial interests or personal relationships which have or could be perceived to have influenced the work reported in this article.

## References

[1] Castillo A., Tabik S., Pérez F., Olmos R., Herrera F. (2019). Brightness guided preprocessing for automatic cold steel weapon detection in surveillance videos with deep learning. Neurocomputing.

[2] Ben Mabrouk A., Zagrouba E. (2017). Spatio-temporal feature using optical flow based distribution for violence detection. Pattern. Recognit. Lett..

[3] Gao Y., Liu H., Sun X., Wang C., Liu Y. (2016). Violence detection using Oriented Violent Flows. Image Vis. Comput..

[4] Dinesh J.S.R., Fenil E., Gunasekaran M., Vivekananda G.N., Thanjaivadivel T., Jeeva S., Ahilan A. (2019). Real time violence detection framework for football stadium comprising of big data analysis and deep learning through bidirectional LSTM. Comput. Netw..

[5] Accattoli S., Sernani P., Falcionelli N., Mekuria D.N., Dragoni A.F. (2020). Violence detection in videos by combining 3D convolutional neural networks and support vector machines. Appl. Artif. Intell..

[6] Bermejo Nievas E., Deniz Suarez O., Bueno García G., Sukthankar R., Real P., Diaz-Pernil D., Molina-Abril H., Berciano A., Kropatsch W. (2011). Violence detection in video using computer vision techniques. Computer Analysis of Images and Patterns.

[7] T. Hassner, Y. Itcher, O. Kliper-Gross, Violent flows: real-time detection of violent crowd behaviour, in: 2012 IEEE Computer Society Conference on Computer Vision and Pattern Recognition Workshops, 2012, pp. 1–6. 10.1109/CVPRW.2012.6239348.

[8] M. Cheng, K. Cai, M. Li, 2019. RWF-2000: an open large scale video database for violence detection, arXiv preprint, arXiv:1911.05913. https://arxiv.org/abs/1911.05913.

